# Unravelling Alveolar Bone Regeneration Ability of Platelet-Rich Plasma: A Systematic Review with Meta-Analysis

**DOI:** 10.3390/bioengineering9100506

**Published:** 2022-09-27

**Authors:** Eduardo Anitua, Mikel Allende, Mohammad Hamdan Alkhraisat

**Affiliations:** 1 Regenerative Medicine Department, BTI Biotechnology Institute, 01007 Vitoria, Spain; 2 Clinical Research, University Institute for Regenerative Medicine and Oral Implantology (UIRMI), 01007 Vitoria, Spain

**Keywords:** alveolar ridge preservation, platelet-rich plasma, platelet rich in growth factors, alveolar bone regeneration

## Abstract

Background: This systematic review aims to evaluate the efficacy of the available platelet-rich plasma (PRP) products and composition to regenerate alveolar bone after tooth extraction. Methods: PubMed, Cochrane Central Register of Controlled Trials, and EBSCO databases were searched up to 2 July 2021. Only randomized clinical trials using leukocyte-rich plasma (L-PRP) or pure-platelet rich plasma (P-PRP) for bone regeneration in alveolar ridge preservation were selected. The following outcomes were considered: (1) new bone formation (primary outcome) and (2) bone density (secondary outcome). A meta-analysis for PRP, P-PRP, and L-PRP using a fixed effect model was performed with Review Manager 5.4 software. Overall evidence was qualified using GRADE. Results: Six randomized clinical trials from 2639 unique articles initially identified met the inclusion criteria. The meta-analysis showed a significant effect of the P-PRP on the outcome of new bone formation (SMD, 1.44; 95% CI, 0.84 to 2.03) for P-PRP treatment. No information was retrieved for L-PRP. A statistically significant difference was also observed in the P-PRP group for bone density outcome (SMD, 1.24; 95% CI, 0.81 to 1.68). The L-PRP treated sockets also showed higher bone density (SMD, 0.88; 95% CI, 0.31 to 1.45) in comparison to control sockets. The quality of evidence was moderate for both outcomes in the P-PRP group and low for the L-PRP group. Conclusions: Despite the limitations of the included studies, our data suggest that P-PRP, in comparison to unassisted healing, can improve alveolar bone regenerative potential. However, more high-quality clinical studies are needed.

## 1. Introduction

Healthy tissue preservation and highly predictable techniques are one the main objectives of the oral implantology. Tooth removal is indicated when it cannot be restored or maintained in acceptable conditions for long-term health, functions, or esthetics. Significant alveolar bone remodeling has been reported after tooth removal [1,2,3,4]. Alveolar socket atrophy may affect tooth replacement therapy, especially when implant-supported restorations are planned. A deficient blood supply and a higher osteoclastic activity may play a pivotal role in this process [5].

Various materials have been used to prevent alveolar bone loss after tooth extraction [6,7]. Generally, these consist of bone graft materials that can be derived from xenogenic [8,9,10], allogenic [11,12], or synthetic materials [13]. Bone derivative materials serve as a scaffold for new bone growth and dimensional stability of the wound [14]. Although good clinical outcomes in terms of bone healing and alveolar socket dimensions have been reported in some clinical trials [6,7], their ability to regenerate hard tissues is still under debate due to a lack of osteoinductive and osteogenic properties [15,16].

Since its discovery, biological agents have emerged as a promising alternative to promote de novo bone formation [17]. Of the available biomaterials, platelet-rich plasma (PRP) has become increasingly popular since its first introduction in the 1990s [18,19]. PRP is characterized by a concentrated autologous solution of platelets obtained using blood gradient density centrifugation. A blood clot is formed after platelet activation, and growth factors and cytokines are released. PRP is used in many medical areas to promote tissue regeneration, including traumatology, dermatology, or oral and maxillofacial surgery. However, the effectiveness of PRP in regenerating alveolar bone tissue remains controversial, mainly due to the different available protocols. The presence or absence of leukocytes is believed to be a key differentiator and has been used to classify PRPs in two main groups: leukocyte-poor PRP (P-PRP) and leukocyte-rich PRP (L-PRP).

Plasma rich in growth factors (PRGF) is a P-PRP that was first described by Anitua [20]. Unlike L-PRP, PRGF is characterized by a relatively modest increase in platelet concentration (≈2–3-fold higher than peripheral blood concentration) and by the absence of leukocytes [21,22]. Several randomized clinical trials [22] (RCTs) have found better soft tissue healing and lower discomfort in PRGF-treated sockets [18,23,24,25,26,27]. It is believed that the growth factors released after platelet activation may also promote osteogenic induction and bone regeneration in the alveolar socket. In this sense, compared to unassisted healing sockets (natural healing or blood clot), a beneficial effect on bone density [18] and a higher new bone formation [27] have been described in the PRGF-treated sockets. By contrast, non-randomized prospective study described no benefit in terms of bone density after 4–8 weeks of follow-up [28].

On the other hand, other findings in RCTs suggest a reduced pain and a higher soft-tissue healing [29] and bone density [30] in the sockets treated with L-PRP. However, several studies have described contradictory results, and the beneficial effect on bone regeneration [29,31] or soft tissue healing [31] could not be confirmed.

Other systematic reviews have assessed platelet concentrates in the context of alveolar bone regeneration [32,33,34]. However, none of them directly compared P-PRP to L-PRP in the context of alveolar bone regeneration, or the information did not contain recently published trials. Thus, this systematic review and meta-analysis investigated the effects of platelet-rich plasma in bone regeneration on alveolar ridge preservation.

## 2. Materials and Methods

### 2.1. Protocol Registration and Reporting Format

The present systematic review was designed following the guidelines of the 2020 Preferred Reporting Items for Systematic Review and Meta-Analysis (PRISMA) statement [35]. The protocol was registered and allocated in the PROSPERO database (CRD42021269495), hosted by the National Institute for Health Research, University of York, Center for Reviews and Dissemination. All amendments performed during the review process were registered indicating the date and the reason for the change.

### 2.2. Focus Question

The aim of this review was to address the following question:

Which platelet-rich plasma shows the best performance for alveolar bone regeneration following tooth extraction?

### 2.3. PICO Strategy

The following Population, Intervention, Comparison and Outcomes framework was used:

- (P) Population: we included patients without a severe underlying disease requiring tooth extraction.
- (I) Interventions: we considered all interventions employing PRP alone for socket filling.
- (C) Comparison: natural healing or blood clot
- (O) Outcome: our primary outcome was new bone formation, whereas we considered bone density as a secondary outcome.

### 2.4. Eligibility Criteria

Included studies were RCTs that met the following criteria: participants requiring tooth extractions, interventions of P-PRP or L-PRP in the alveolar socket, the comparator was unassisted socket healing, and outcomes were new bone formation measured by histomorphometric analysis and bone density. The outcome measurement was limited to 3 months, and there was no limit on the number of patients treated. We excluded observational studies and trials with inadequate information or data.

### 2.5. Data Sources and Search Strategy

**Figure 1:**
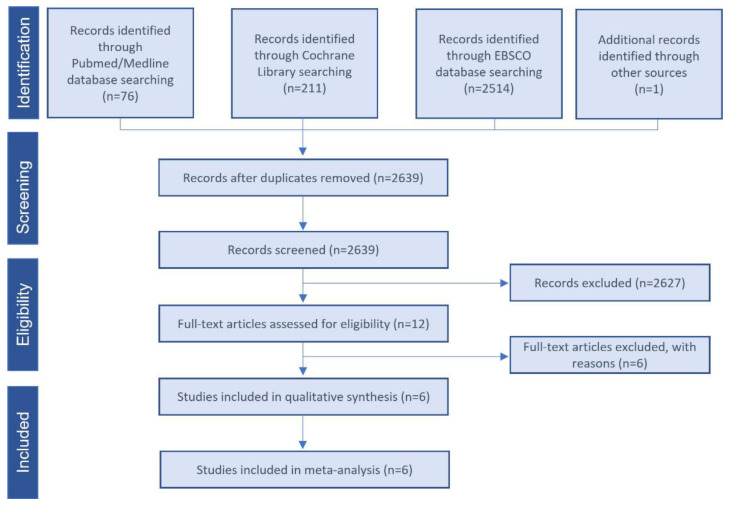
Study selection flow diagram. PRISMA flow diagram of the screening and selection process.

A comprehensive electronic search was performed from inception to 2 July 2021 using the following internet databases: MEDLINE/Pubmed, Cochrane Central Register of Controlled Trials, and EBSCO. Using controlled vocabulary supplemented with keywords, we searched for RCTs using PRP for alveolar ridge preservation. The search strategy used the following terms: (alveolar ridge preservation OR tooth extraction OR socket) AND (platelet rich plasma OR platelet-rich plasma OR platelet rich fibrin OR platelet concentrate). In addition, we searched clinical trial registries (http://www.clinicaltrials.gov) to find studies in the grey literature. The database was accessed on 2 July 2021. No language restriction was applied in the search process. The reference lists of related systematic reviews were also reviewed to identify possible additional studies. The search was limited to human studies. Two reviewers (MA and MHA) independently screened the title and abstract of each publication to exclude any that did not address the research question of interest. The same reviewers evaluated the remaining articles’ full texts to identify studies that met all criteria for inclusion in the quantitative meta-analysis. Figure 1 details the study selection flowchart.

### 2.6. Data Collection and Management

MA and MHA independently screened the titles and abstracts of the articles identified in the search. In case of disagreement, a consensus was reached by discussion. Next, the full text of all qualified studies or for which there was insufficient information in the title and abstract to make a decision were obtained. Subsequently, the same two reviewers (MA and MHA) independently checked the full text publications for inclusion. Any discrepancies were resolved by an open discussion between reviewers. Studies that did not meet the inclusion/exclusion criteria were excluded, and the reason for exclusion is indicated in Table S1.

### 2.7. Data Extraction

MA independently abstracted all relevant data from the included articles onto a specific spreadsheet. The following characteristics were recorded: (a) study characteristics—primary author, time period of study/year of publication; (b) RCT design characteristics—split-mouth, parallel; (c) patient characteristics—age, sex, and number of tooth extractions or localization; (d) follow-up period; (e) outcome assessment—histology evaluation technique, bone density measurement methodology; (f) intervention groups—control and experimental groups. MA entered data into Review Manager 5.4 and double checking was performed for accuracy.

### 2.8. Risk of Bias in Individual Research Studies

We assessed risk of bias of included studies following the recommendations of Cochrane Handbook for Systematic Reviews of Interventions [36]. Using these standardized criteria, two authors (MA and MHA) judged the risk of bias across each study in the six following domains: random sequence generation, allocation concealment, blinding of participants and personnel, blinding of outcome assessment, incomplete outcome data, and selective reporting. Any discrepancies in judgements of risk of bias were resolved by open discussion. Once these domains were assessed, an overall rating was assigned to each study. The overall score was low risk when none of the six domains were found to be at high risk and if three or less domains were found to be at unclear risk. A moderate risk was assigned when one domain was found to be at high risk; or no domains were found to be a high risk but four or more were found to be at unclear risk. In all other cases, the publication was classified as overall high risk of bias.

### 2.9. Outcomes

The primary outcome was new bone formation in the extraction socket, measured as the mean value using histomorphometry analysis. New bone formation was defined according to the study authors; if this was not clearly defined, it was classified based on the presence of mineralized tissue area. Our secondary efficacy outcome was alveolar bone density after the observation period. It was defined as the radiodensity obtained after evaluating alveolar bone in periapical radiographs, cone beam computed tomography (CBCT), or micro-CT. If the data were missing or unclear, attempts were made to contact the corresponding author for additional information or clarification.

### 2.10. Statistical Analysis

The data for the quantitative assessment were extracted for the primary and secondary outcomes and subjected to meta-analysis. The software Review Manager 5.4 (The Nordic Cochrane Centre, Copenhagen, Denmark) was used to perform the meta-analysis using the inverse variance method. For continuous data outcomes, such as new bone formation or bone density, the standard mean difference (SMD) and 95% confidence intervals (CI) were calculated. SMD was selected as the metric of choice because the measurement units were not the same. The effect was considered significant when *p* ≤ 0.05. Heterogeneity was assessed using the I^2^ statistic, with values over 50% indicating substantial heterogeneity [37]. The fixed-effects model was used when no significant heterogeneity was found (I^2^ ≤ 50%), whereas the random-effects model was adopted when a significant heterogeneity (I^2^ > 50%) was detected. The results were shown in a forest plot to provide a graphical overview of the data. If possible, a sub-group analysis was performed according to the type of PRP used (L-PRP or PRGF). Meta-analyses were performed only for studies with comparable outcome measures and observation times. Due to the low number of studies, sensitivity analyses and reporting bias assessment using funnel plots could not be performed.

### 2.11. Certainty of Evidence

Two authors (MA and MHA) independently evaluated the overall certainty of evidence. The Grading Recommendation, Assessment, Development, and Evaluation (GRADE) approach [36] was followed to assess the certainty of evidence of primary and secondary outcomes of the studies included in the meta-analyses. We judged the certainty of evidence as high, moderate, low, or very low. GRADEpro Guideline Development Tool (McMaster University, University, 2020, developed by Evidence Prime, Inc., available from gradepro.org) was used to assess the quality of the body of evidence [38]. The decisions to down- or upgrade the level of certainty were justified.

## 3. Results

### 3.1. Study Selection and Characteristics

From a total of 2639 unique publications identified in the electronic databases using our search strategy, we retrieved 12 publications, and finally included six studies (Figure 1). Supplementary Table S1 lists the excluded studies and the reasons for exclusions. We excluded studies because an unassisted socket healing arm was not included, the design of trial was not an RCT, or different outcomes were reported. The six selected clinical trials were dated from 2010 to 2020 and included 188 participants (range 18–74 years). All trials were randomized, blinded for the participant and controlled for unassisted socket healing. The follow-up period to assess new bone formation and bone density was 8–10 weeks and 10–12 weeks, respectively.

The main characteristics of the selected RCTs are summarized In Table 1. Two trials used a split-mouth design, whereas a parallel design was employed in four studies. Overall, 229 teeth were extracted in 188 subjects. L-PRP and P-PRP were applied in 27 and 76 sockets, respectively. The primary outcomes of new bone formation and bone density are indicated in Table 2 and Table 3, respectively. Three studies presented new bone formation data, whereas four studies reported alveolar bone density information.

### 3.2. Risk of Bias of Included Trials

**Figure 2:**
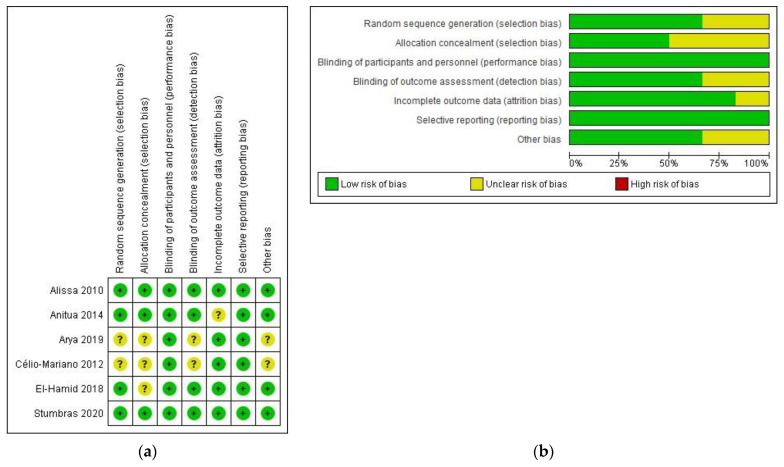
Study selection flow diagram. PRISMA flow diagram of the screening and selection process. Quality assessment of the included RCTs. (**a**) Risk of bias summary: review authors’ judgments about each risk of bias item for each included study: (+), low risk of bias; (?): unclear risk of bias. (**b**) Risk of bias graph: review authors’ judgments about each risk of bias item presented as percentages across all included studies.

The risk of bias assessment is summarized in Figure 2. Three trials clearly described the allocation concealment procedure and were judged as low risk of bias. The rest did not mention the allocation in the text and were classified as unclear risk of bias. Due to the characteristics of the outcomes, blinding of participants and personnel domain was scored as low risk of bias. Four studies were blinded for outcome assessment and judged as low risk of bias. Blinding to outcome assessment was unclear in two publications, as a clear description was not provided. The follow-up reports were completed for five trials, with one trial classified as unclear risk of bias. Overall, four studies were classified as low risk, and two trials were scored as having moderate risk.

### 3.3. Primary Outcome: New Bone Formation

**Figure 3:**
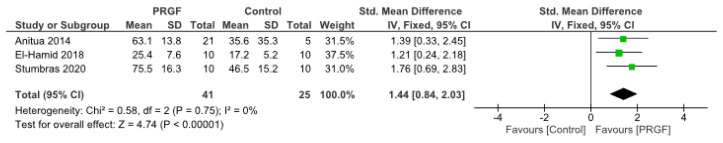
Meta-analysis of the studies evaluating new bone formation. SE: standard error; SMD: standardized mean difference; CI: confidence interval.

Three studies evaluated the performance of PRGF in new bone formation using histomorphometric analysis, whereas we could not find any study employing L-PRP (Table 2). Bone biopsy specimens (25 controls and 41 PRGF) were harvested at 8–10 post-operative weeks and stained with hematoxylin and eosin and May–Grünwald–Giemnsa [18,39], or Masson’s Trichrome [40]. Meta-analysis revealed that new bone formation was statistically higher for sockets treated with PRGF (SMD, 1.44; 95% CI, 0.84 to 2.03) (Figure 3). In addition, the consistency among studies was high (heterogeneity index I^2^ = 0%, *p* = 0.93).

### 3.4. Secondary Outcome: Bone Density

**Figure 4:**
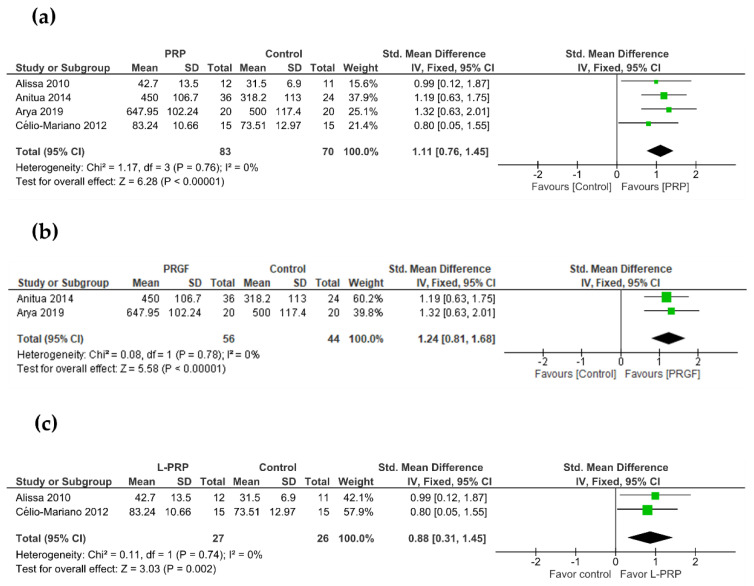
Meta-analysis of the studies evaluating bone density. (**a**) PRP, (**b**) PRGF, (**c**) L-PRP. SE: standard error; SMD: standardized mean difference; CI: confidence interval.

In total, 4 out of 6 studies evaluated alveolar bone density after a follow-up period of 10–12 weeks (Table 3). PRGF and L-PRP were present in two trials each. In two studies, radiographic density was measured using CBCT [18,27]. Two other trials measured trabecular bone volume (%) [29] and relative bone density (%) [41] with radiographic assessment. Regardless of the type of PRP, the meta-analysis reported a higher bone density in treated sockets in comparison to control sockets (SMD, 1.11; 95% CI, 0.76 to 1.45; I^2^ = 0%) (Figure 4a). When a subgroup analysis was performed, PRGF significantly increased bone density over unassisted healing sockets (SMD, 1.24; 95% CI, 0.81 to 1.68; I^2^ = 0%) (Figure 4b). L-PRP also showed a positive effect on the alveolar bone density (SMD, 0.88; 95% CI, 0.31 to 1.45; I^2^ = 0%) (Figure 4c).

### 3.5. Quality of Evidence

Overall evidence was qualified using GRADE for all evaluated outcomes. Regarding to the studies with PRGF, the quality of evidence for new bone formation and bone density was judged as moderate. With regard to L-PRP studies, bone density was scored as low. The reasons for a downgrade/upgrade of the quality of evidence are indicated in Table 4 and Table 5.

## 4. Discussion

PRP preparations have been widely used in the oral and maxillofacial field in recent decades to promote tissue regeneration and prevent post-operative discomfort [42,43]. However, the high heterogeneity among PRP preparation protocols has resulted in platelet concentrate products that differ significantly in composition and functions [44]. Although the main PRP groups (L-PRP and PRGF) have been proven to be effective in socket preservation [18,27,29,30], evidence from clinical trials suggests more consistent and reproducible data when there is a lack of leukocytes in the preparation [22].

PRP has been associated with better healing in several studies, including a beneficial effect on soft-tissue healing and regeneration [45], new capillary growth promotion [46], or acceleration of epithelization in the chronic wound [47]. As these beneficial effects of platelet concentrate products are caused by the release of growth factors during the first days, it is still debatable whether PRP may stimulate bone healing. The aim of this systematic review was to investigate the effects of platelet-rich plasma in bone regeneration in alveolar ridge preservation.

A total of six articles were selected and analyzed in this systematic review. Our study employed new bone formation as the primary outcome to evaluate the bone-regeneration ability of the PRP. New bone formation information was presented in three studies. Bone density was the secondary outcome and data were found in four studies. Given the small number of the eligible studies, the results should be interpreted with caution. Nonetheless, we observed a very low heterogeneity among the included studies, indicating a high-standard design and a low source of bias. In line with this, most of the studies of our systematic review were judged as having medium-to-high quality based on the criteria set for the risk of bias assessment.

PRGF is considered a type of platelet-rich plasma characterized by a complete lack of leukocytes [20]. Unlike other PRP, this product is prepared following a reference protocol that did not undergo major modifications since its introduction [34], facilitating the interpretation and reproducibility of the clinical data. The results from our meta-analysis seemed to show significantly greater new bone formation in the PRGF group compared with unassisted healing. Unfortunately, no data were found for the L-PRP treatment, and a quantitative analysis could not be performed for this group. Our analysis was in accordance with another systematic review that assessed the efficacy of different graft materials in alveolar socket preservation [32]. Anitua and colleagues have reported the ability of PRGF to regenerate hard tissue in post-extraction sockets by histomorphometric analysis [18]. However, in the same systematic review the authors expressed their concerns due to the limited number of biopsies analyzed in the unassisted healing group (*n* = 5) [32]. Recently, two independent RCTs have reproduced these observations by employing the same preparation protocol and using a comparable follow-up time and methodology, providing strong evidence on using PRGF for alveolar bone regeneration in clinical practice [39,40].

Furthermore, our quantitative meta-analysis revealed a significantly higher bone density in PRP sockets than in unassisted socket healing. Different studies have employed different techniques to quantify bone density (periapical radiographs or cone-beam CT scan (CBCT)). CBCT might be a more sensitive technique than periapical radiographs in the measurement of changes in the radiographic bone density. A recent in vitro study suggested that traditional periapical images might not be sensitive enough to detect density variation in the range of bone mineral density [48]. Therefore, future well-designed trials should include high-sensitive techniques such as CBCT in order to analyze alveolar density changes properly.

A systematic review dated 2017 investigated socket preservation of different platelet concentrates in terms of healing, probing depth, and bone density [34]. According to their results, PRGF and L-PRP showed a better performance regarding the outcome of bone density. However, it must be noticed that their analysis included prospective studies along with RCTs, introducing an important source of bias with the selection of non-randomized studies. Moreover, their results are based on two studies (one PRGF and one L-PRP study); therefore, their conclusions should be interpreted cautiously. In comparison to the previous review, our review included three new studies that have been recently published. We believe that our study can provide new insights into hard tissue healing after tooth removal.

Healing is affected by many factors that can make the interpretation complex. Indication for tooth extraction is a possible confounding factor which may have a relevant influence on socket healing. Five trials included the reasons for tooth extraction in the publication, and only one excluded patients with significant periapical or periodontal disease. Future studies should be more rigorously designated to avoid a misinterpretation of the results.

Another characteristic that can severely affect the ability of PRP to preserve alveolar tissue is the participants’ smoking habits. Smoking is believed to be associated with delayed wound healing, and a significant reduction in alveolar width and bone density in the alveolar sockets of patients that smoke [49,50]. Unreliable results regarding hard-tissue healing have been obtained when an unusual percentage of subjects was present in one of the experimental groups [28]. Two of our selected studies excluded cigarette-smoking subjects from the trials, two studies presented smoking participants homogenously distributed across the groups, and two trials did not include information regarding the smoking habit. Appropriate inclusion/exclusion criteria in the trial design stage is of critical importance to evaluate alveolar bone regeneration ability, and future studies should take this into account.

Several limitations affect the present work. No distinction was made between mandibular or maxillary tooth extraction sites [29,40,41]. Furthermore, several studies only considered a specific tooth type such as third molar [41], molar [18], or premolar sites [40]. The selection of participants from different populations might be an important source of bias. For that, a complete description of demographic and clinical information should be provided to avoid confounding factors. In addition, the low number of studies evaluating the different PRP products would indicate the need for more new studies. However, this limitation was reduced with the exclusive inclusion of randomized clinical trial and the selection of trials with similar follow-up times or methodology.

## 5. Conclusions

Despite the limitations of the included studies, our data suggest that PRGF, in comparison to unassisted healing, can improve alveolar bone regenerative potential (new bone formation and bone density). Unfortunately, the effect of L-PRP could not be assessed for the primary outcome (new bone formation) However, more high-quality clinical studies are needed.

## Figures and Tables

**Table 1:** Main characteristics of the included studies.

Study	Design	Patients (Teeth)	Sex Male/Female	Age Years	Site Characteristics	Follow-Up	PRP Preparation Protocol	Intervention	
								Control	Test
Stumbras et al., 2020 [39]	RCT ^1^	40	14/26		Anterior maxilla	12 weeks	580 g; 8 min	Natural healing	PRGF
Alissa et al., 2010 [29]	RCT	23 (29)	8/7	20–52	Mandible or maxilla	12 weeks	3200 rpm; 12 min	Non-PRP	L-PRP
Arya et al., 2019 [27]	Split mouth RCT	20 (40)	13/7	15–30	Mandible	13 weeks	580 g; 8 min	Empty socket	PRGF
Célio-Mariano et al., 2012 [40]	Split mouth RCT	15 (30)	7/8	18–22	3ºmolars	6 months	160 g; 20 min 400 g; 15 min	Blood clot	L-PRP
Anitua et al., 2015 [18]	RCT	60	29/31	18–74	Molar extraction in the mandible	12 weeks	580 g; 8 min	Blood clot	PRGF
El-Hamid et al., 2018 [41]	RCT	30	6/24	C:30.1 ± 7.5 T: 29.2 ± 4.4	Premolars	2 months	580 g; 8 min	Natural healing	PRGF

^1^ Additional experimental groups are present, NR: not reported; PRP: platelet-rich plasma; PRGF: platelet-rich in growth factors.

**Table 2:** Histomorphometric analysis of the included studies.

Study	Time of Measurement	Sample Size	Staining	Histomorphometric Analysis	
		C/T		Control	Test
Stumbras et al., 2020 [39]	12 weeks	10/10	May Grünwald-Giemsa	New formed mineral tissue (%) 46.5 ± 15.2	New formed mineral tissue (%) 75.5 ± 16.3
Anitua et al., 2015 [18]	10–12 weeks	5/21	HE and MGG	New bone regeneration (%) 35.6 ± 35.3	New bone regeneration (%) 63.1 ± 13.8
El-Hamid et al., 2018 [41]	8 weeks	10/10	Masson’s Trichrome	Mineralized tissues (%) 17.2 ± 5.2	Mineralized tissues (%) 25.4 ± 7.6

HE: hematoxylin–eosin; MGG: May–Grünwald–Giemsa.

**Table 3:** Bone density measurements of the included studies.

Study	Time of Measurement	Sample Size	Method	Bone Density	
		C/T		Control	Test
Alissa et al., 2010 [29]	12 weeks	8/8	Periapical radiographs	Trabecular bone volume (%) 31.5 ± 6.9	Trabecular bone volume (%) 42.7 ± 13.5
Arya et al., 2019 [27]	13 weeks	20/20	CBCT	Mean bone density (HU) 500.05 ± 117.40	Mean bone density (HU) 647.95 ± 102.24
Célio-Mariano et al., 2012 [40]	3 months	15/15	Periapical radiographs	Mean bone density (%) 73.51	Mean bone density (%) 83.24
Anitua et al., 2015 [18]	10–12 weeks	22/30	CBCT	Mean bone density (HU) 318.2 ± 113.0	Mean bone density (HU) 450.0 ± 106.7

HU: Houndsfield units; CBCT: Cone Beam Computed Tomography.

**Table 4:** Summary of the quality assessment using the GRADE approach of outcomes included in the meta-analysis of PRGF.

Certainty Assessment							№ of Patients		Effect		Certainty	Importance
No of Studies	Study Design	Risk of Bias	Inconsistency	Indirectness	Imprecision	Other Considerations	[Intervention]	[Comparison]	Relative (95% CI)	Absolute (95% CI)		
New bone formation (assessed with: histomorphometry)												
3	Randomized trials	Not serious	Not serious	Not serious	Serious ^a^	Publication bias strongly suspected ^b^ Strong association ^c^	41	25	-	SMD 1.44 SD higher (0.27 higher to 2.6 higher)	⨁⨁⨁◯ Moderate	CRITICAL
Bone density (assessed with: CBCT)												
2	Randomized trials	Not serious	Not serious	Not serious	Serious ^a^	Publication bias strongly suspected ^b^ Strong association ^c^	50	42	-	SMD 1.24 SD higher (0.39 higher to 2.09 higher)	⨁⨁⨁◯ Moderate	CRITICAL

CI: confidence interval; RCT: randomized trials; SMD: standardized mean difference. ^a^. imprecision judged as serious due to the low number of included studies. ^b^. the possibility of publication bias cannot be excluded, thus, we downgraded by one level the quality of evidence. ^c^. the effect was considered large if at least 2 studies showed an SMD > 0.8.

**Table 5:** Summary of the quality assessment using the GRADE approach of outcomes included in the meta-analysis of L-PRP.

Certainty Assessment							№ of Patients		Effect		Certainty	Importance
No of Studies	Study Design	Risk of Bias	Inconsistency	Indirectness	Imprecision	Other Considerations	[Intervention]	[Comparison]	Relative (95% CI)	Absolute (95% CI)		
Bone density (assessed with: periapical radiography)												
2	RCT	Not serious	Not serious	Not serious	Serious ^a^	Publication bias strongly suspected ^b^	23	23	-	SMD 0.88 SD higher (0.24 lower to 2 higher)	⨁⨁◯◯ Low	CRITICAL

CI: confidence interval; RCT: randomized trials; SMD: standardized mean difference. ^a^. imprecision judge as serious due to the low number of included studies. ^b^. the possibility of publication bias cannot be excluded, thus, we downgraded by one level the quality of evidence.

## Data Availability

Data supporting the findings are available within the article and from the authors upon request.

## Supplementary Materials

The following supporting information can be downloaded at: https://www.mdpi.com/article/10.3390/bioengineering9100506/s1, Table S1: List of excluded studies with reasons for exclusion
