# Supplementary material for: Unravelling Alveolar Bone Regeneration Ability of Platelet-Rich Plasma: A Systematic Review with Meta-Analysis
Source: Bioengineering (Basel). 2022 Sep 27;9(10):506. doi: 10.3390/bioengineering9100506 (PMC9598440; doi:10.3390/bioengineering9100506)
Supplement: Supplementary file 1 [file bioengineering-09-00506-s001.zip › bioengineering-1849885-Supplementary.pdf]

Supplementary Table S1. List of excluded studies with reasons for exclusion.

Excluded study	Reason for exclusion
Sammartino et al., 2005	No controlled arm
Stumbras et al., 2021	Different outcomes
Farina et al., 2013	Non-randomized clinical trial
Nisar et al., 2020	Different outcomes
De Antonello et al., 2013	Non-randomized clinical trial
Rutkowski et al., 2010	Non-randomized clinical trial
